# Prevalence and Outcome of Delirium in Proximal Femur Fracture Patients Undergoing Orthogeriatric Treatment

**DOI:** 10.3390/jcm15176842

**Published:** 2026-09-03

**Authors:** Vanessa Ketter, Benjamin Buecking, Daphne Asimenia Eschbach, Ahmad Alterk, Steffen Ruchholtz, Carsten Schoeneberg, Bastian Pass, Hannah Schmidt, Tom Knauf

**Affiliations:** 1 Helios Kliniken Kassel, 34121 Kassel, Germany; 2 Center for Orthopaedics and Trauma Surgery, University Hospital Giessen and Marburg GmbH, Philipps-University Marburg, Location Marburg, 35043 Marburg, Germany; 3 Department of Orthopedic and Emergency Surgery, Alfried Krupp Hospital, Hellweg 100, 45276 Essen, Germany; 4 AUC—Academy for Trauma Surgery, 80538 Munich, Germany

**Keywords:** geriatric, delirium, proximal femur fractures

## Abstract

**Background**: Geriatric trauma centers (ATZ) in Germany provide interdisciplinary care for elderly trauma patients with the objective of enhancing treatment outcomes following proximal femur fractures. Delirium, a prevalent and grave complication in this patient population, has been associated with elevated mortality and functional limitations. The objective of this study was to examine the prevalence and consequences of delirium in proximal femur fractures by utilizing the Registry for Geriatric Trauma (ATR-DGU). **Methods**: The study encompassed 32,934 patients aged ≥70 years who sustained proximal femoral fractures during the period 2022–2023. The demographic and clinical parameters, fracture type, time of surgery, mobility, complications, and mortality were meticulously documented. The primary endpoint of the study was the occurrence of delirium and its influence of the outcome of hip fracture patients. Additionally an analysis was conducted to account for peri-implant/periprosthetic fractures. **Results**: Of the 21,127 patients who underwent screening, 23.6% had already been diagnosed with delirium upon admission. The delirium rate did not differ between hip-/pertrochanteric and peri-implant/periprosthetic fractures. Patients in a delirious state were found to be older, frailer, and more dependent on care. They exhibited more non-surgical complications, poorer mobility, and more frequent transfers to nursing homes. In the context of peri-implant/periprosthetic fractures, delirium showed no significant difference regarding surgical revision (*p* = 0.192) and mortality rates (*p* = 0.229). **Conclusions**: Delirium is therefore a prevalent and clinically significant complication. Its early detection and prevention are essential for proximal femur fracture patients in orthogeriatric care.

## 1. Introduction

Since 2014, Germany has had geriatric trauma centers (ATZ) that specialize in the treatment of orthogeriatric patients. The ATZs offer comprehensive care for older adults (≥70 years) following accidents or falls, often through the collaborative efforts of an interdisciplinary team of physicians, physical therapists, psychologists, and other specialists. These professionals collaborate to develop an individualized treatment plan for each patient. This plan is designed to address the unique needs of older patients, including their pre-existing health conditions, mobility, and living circumstances.

Delirium has been identified as a serious complication in the inpatient treatment of elderly patients over 65 years of age. This condition has been associated with an increased risk of mortality, prolonged hospital stays, permanent cognitive impairment, and nosocomial infections [1].

Proximal femur fractures are associated with a high postoperative morbidity rate, reduced quality of life, high mortality rates, and limited mobility and independence [2,3].

Delirium has been demonstrated to exert an additional deleterious effect on the clinical course in patients who have sustained fractures in proximity to the hip joint [4].

Preoperative delirium has been demonstrated to be a risk factor for delayed surgery [5,6]. This association suggests a heightened probability of postoperative delirium, which has been found to be associated with an increased risk of mortality, loss of function, and institutionalization [7,8,9].

Despite the implementation of orthogeriatric co-management and the objective of optimizing surgical care and long-term rehabilitation measures, the treatment of patients with proximal femur fractures remains challenging.

It can be hypothesized that the collaborative management of patients through trauma surgery and geriatric treatment during hospitalization can result in a reduction in delirium rates [10].

The efficacy of co-management in reducing delirium and enhancing patient outcomes has been a subject of considerable interest in the medical literature. While the impact of delirium on the severity of in-hospital complications remains a contentious issue, there is a growing consensus that co-management can mitigate the adverse effects of delirium, thereby improving patient well-being and outcomes.

This prompts the investigation of the potential benefits of trauma surgery and geriatric co-management in geriatric trauma centers, including the reduction in delirium rates, minimization of complications, and enhancement of patient outcomes.

## 2. Materials and Methods

The “Registry for Geriatric Trauma” (AltersTraumaRegister DGU^®^, ATR) was founded in 2016 by the German Trauma Society (DGU). The aim of the ATR-DGU is the pseudonymized and standardized documentation of data on the situation prior to the accident, the diagnosis, the treatment and the outcome in geriatric patients with proximal femur fractures. Therefore, all DGU-certified “AltersTraumaZentren” (Specialty Orthogeriatric Departments) are required to enter patient data into the ATR-DGU. Afterwards, patient data are transferred pseudonymized via a web-based application into a central database. Currently, about 170 hospitals from Germany, Switzerland, Austria and Luxembourg contribute to the ATR-DGU. The scientific management of the ATR-DGU is carried out by the Working Committee on Geriatric Trauma Registry (AK ATR) of the DGU. Approval for scientific data analysis from the ATR-DGU is granted via a peer-review process in accordance with the publication guidelines laid out by the AK ATR. The present study is in accordance with the publication guidelines of the ATR-DGU and registered as ATR-DGU project ID 2024-001. A favorable ethical approval (AZ46/16) has been granted by the Ethics Committee of the Philipps University of Marburg.

The inclusion criteria of the ATR-DGU are patients with proximal femur fractures, including periprosthetic and peri-implant fractures, requiring surgery and who are aged 70 years or older. The ATR-DGU collects data in five distinct phases: admission, pre-surgery, surgery, first week post-surgery, discharge/transfer and an optional 120-day follow-up.

This study analyzed the data of 32,934 patients entered into the registry between 2022 and 2023.

In this study, we investigated the occurrence of delirium in patients with hip-related fractures and its impact on the outcome of these patients in an orthogeriatric setting. In addition, we subdivided the entire group based on fracture type into another study cohort with periprosthetic and peri-implant fractures, in which we examined the same parameters. Our baseline data included the following items: age, gender, “American Society of Anaethesiologists” (ASA) Score, time to surgery, delirium screening at the moment of admission and the existence of delirium during hospitalization, “Clinical Frailty Scale” (CFS), “Identification of seniors at risk” (ISAR) score, residential status, other fractures, walking ability (before fracture, on the 7th day after surgery and follow-up), length of stay, type of fractures, occurrence of delirium during hospitalization, surgical complication (at the initial stay and follow-up), discharge after hospital and current location at the follow-up, and mortality (initial stay and follow-up). The diagnosis of delirium was made either on the basis of the Nu-Desc score or another delirium screening tool.

### Statistical Analysis

All calculations were performed using statistics software R v. 4.0.2 (Foundation for Statistical Computing, Vienna, Austria). For descriptive analyses, categorical data were presented as counts and percentages and continuous variables as medians with interquartile range (IQR). Variables were compared between the groups (delirium versus no delirium at admission and delirium versus no delirium during hospitalization) using the Χ^2^-test for categorical variables and the Wilcoxon test for continuous variables. Differences were considered statistically significant when *p* < 0.05.

To assess the association between the groups and different outcome parameters, multivariable regression analyses were performed. Depending on the distribution and type of the outcome variable, either binary logistic regression or linear regression models were applied. Continuous outcome variables were logarithmically transformed to improve model assumptions. All regression models were adjusted for age, sex, ASA classification, pre-fracture walking ability, and ISAR Score (0–1 vs. ≥2). Analyses investigating delirium during hospitalization were additionally adjusted for fracture type. Results from linear regression analyses are presented as percentage change with 95% confidence intervals (95% CI), while results from logistic regression analyses are presented as odds ratios (OR) with 95% CI.

## 3. Results

### 3.1. Delirium by the Time of Admission to the Emergency Department

**Figure 1:**
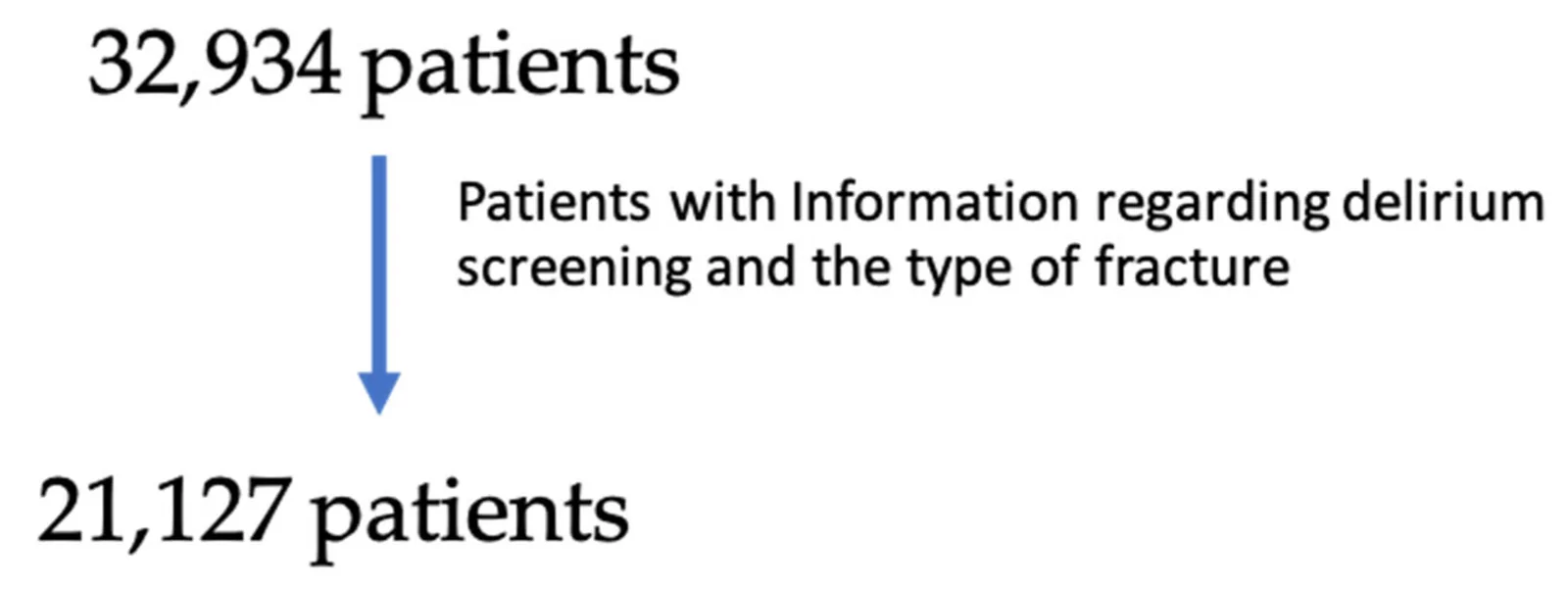
Flow chart.

A total of 32,934 patients from 163 hospitals were entered into the register between 2022 and 2023. Information on delirium screening and fracture type was available for 21,127 patients (Figure 1).

No clinically relevant differences were observed in the baseline characteristics between the included patients and those who had to be excluded due to a lack of information regarding delirium screening and the type of fracture.

A total of 4991 (23.6%) patients were already suffering from delirium when they were admitted to the emergency department, 19,883 suffered from either a pertrochanteric/subtrochanteric or a femoral neck fracture, and 1244 patients suffered from a periprosthetic/peri-implant fracture. There was no difference between the occurrence of delirium upon admission to the emergency department between these two groups 23.6% (periprosthetic/peri-implant fracture n = 294; proximal femur fracture n = 4697).

### 3.2. Proximal Femur Fracture Patients

Patients with proximal femur fractures and additionally suffering from delirium upon admission are older, at 86.0 years (interquartile range (IQR) 82–91) vs. 84.0 years (IQR 80–88), have a high ASA Score, 3.0 (IQR 3.0–3.0) vs. 3.0 (IQR 2.0–3.0), and are more often living in a nursing home (38.4% vs. 13.4%) compared to proximal femur fracture patients not suffering from delirium. These differences can be shown in the ISAR Score (ISAR ≥ 2; 93.2% vs. 75.4%) and CFS 6.0 (IQR 5.0–7.0) vs. 4.0 (IQR 3.0–6.0) as well. Patients with delirium upon admission are more often treated by a geriatrician preoperatively (36.8% vs. 26.6%). These patients are slightly more often transferred to the Intensive Care Unit postoperatively (28.8% vs. 27.3%). A higher mortality rate is observed in the group of patients who were already experiencing delirium at admission (OR 1.35, *p* < 0.001) and the worse mobility in proximal femur fracture patients. These patients are more often discharged to a nursing home (45.5% vs. 21.8%). The length of stay is shorter in patients suffering from delirium upon admission at 13.1 days (IQR 8.1–20.0) vs. 16.1 (IQR 9.1–20.1). There is no significant difference regarding the operational revision rate (2.5% vs. 2.9%). Further information is shown in Table 1.

In addition, a multivariate analysis was performed. This revealed that, after adjustment, patients with delirium at admission had, on average, the same preoperative length of stay—with a 95% confidence interval of −0.32% to 4.37%—compared to the reference group without delirium at admission (*p* = 0.878).

Furthermore, patients with delirium at admission had, on average, a 12.3% shorter total length of stay, with a 95% confidence interval of −14.96% to −9.54%, compared to the reference group without delirium at admission. The difference is statistically significant (*p* < 0.001). The remaining results can be found in Table 2.

### 3.3. Patients with Periprosthetic Fracture

Similar to the results for proximal femur fractures, patients with periprosthetic/peri-implant fractures who already had delirium upon admission are older (86 years (IQR 83–90) vs. 84 years (IQR 79–88)), have a high ASA Score, 3.0 (IQR 3.0–3.0) vs. 3.0 (IQR 3.0–3.0), and are living more often in a nursing home (28.7% vs. 10.4%) compared to patients not suffering from delirium. This difference can also be reflected in the ISAR Score (ISAR ≥ 2; 91.6% vs. 78.9%) and CFS 6.0 (IQR 5.0–7.0) vs. 4.0 (IQR 3.0–6.0).

Patients with delirium upon admission are more often seen by a geriatrician preoperatively (42.4% vs. 34.2%). Additionally, those patients are more often transferred to the Intensive Care Unit postoperatively (50.7% vs. 40.1%).

The univariate analysis indicated an influence of delirium present at admission to be associated with an increased mortality (9.6% vs. 4.1%), poorer mobility, a higher rate of transfer to nursing homes, and an increased rate of surgical revision procedures (9.6% vs. 5.7%). However, the multivariate analysis performed did not reveal any statistical significance for these parameters.

The multivariate analysis showed that patients with delirium at admission had, on average, a 20.5% shorter preoperative length of stay, with a 95% confidence interval ranging from −33.5% to −5.1%, compared to the reference group without delirium at admission (*p* = 0.011).

In addition, patients with delirium at admission had, on average, a 12.2% shorter total length of stay, with a 95% confidence interval ranging from −20% to −3.7%, compared to the reference group without delirium at admission. The difference is statistically significant (*p* = 0.006). Further information is shown in Table 3 and Table 4.

### 3.4. Occurrence of Delirium During Hospitalization

In addition, we examined the incidence of delirium during the hospital stay of patients who did not suffer from delirium upon admission regardless of fracture type. Patients with delirium at admission were excluded in the analysis.

During their hospital stay, 8.3% of the patients with proximal femur fracture and 9.8% of the patients with periprosthetic/peri-implant fracture developed delirium.

The multivariate analysis revealed no statistical significance regarding mortality during the hospital stay or at the 120-day follow-up. Further information are shown in Table 5 and Table 6.

## 4. Discussion

The aim of this study was to investigate the incidence of delirium upon admission to the emergency department in hip fracture patients. We also examined the risk of developing delirium during hospitalization in this patient group. Furthermore, we investigated the influence of delirium on the further course of treatment and mortality. We performed an additional analysis between patients with femoral neck fractures/pertrochanteric fractures and patients with periprosthetic fractures.

The data set used in this study was derived from the registry of geriatric trauma for the 2022–2023 period. Upon admission, patients were classified into two distinct groups: those who suffered from delirium and those who did not suffer from delirium. Additionally, patients were categorized based on the type of fracture sustained, whether it was a proximal femur fracture or a periprosthetic fracture.

This analysis from the Registry for Geriatric Trauma (ATR-DGU) confirms that delirium is a prevalent and clinically significant complication in geriatric patients with proximal femoral fractures. The comparable delirium rate between proximal femur fractures and peri-implant/periprosthetic fractures indicates that the type of fracture itself is not the primary factor. Rather, patient-related factors such as age, frailty, and need for care appear to be more significant.

A review of the literature pertaining to the subject was conducted in the S3 guideline “Comprehensive Geriatric Assessment (CGA) in Hospitalized Patients,” which was published by the German Society for Geriatrics. The results of this review demonstrated that CGA can serve as a preventative measure against delirium [11]. A Cochrane Review found a significant reduction in the risk of delirium by 25% [95% CI 6; 40] in proximal femur fractures [12]. This finding was corroborated by another meta-analysis, which documented a substantial risk reduction of 19% [95% CI 6; 31] [13]. However, the available evidence suggests that there are no significant differences in terms of the rate, duration, or severity of delirium episodes [14]. The extant literature has not provided any evidence that CGA could be deliriogenic [11].

At admission, approximately 24% of all patients screened in this study exhibited signs of delirium, thereby emphasizing the elevated vulnerability of this patient population. Upon admission, patients with delirium were, on average, older (mean age: 86 years), had a higher level of comorbidity (ASA III: 72%), and were more likely to have been residing in nursing homes (38% vs. 13%). As demonstrated in the extant literature, advanced age is a risk factor for the development of delirium during hospitalization [15]. Additionally, higher ASA, institutionalization, comorbidities, and pre-existing dementia have been shown to have a negative impact [16,17].

The majority of studies on delirium in proximal femur fractures have focused on postoperative delirium [18,19], while only a few have addressed preoperative delirium. Previous studies have reported a preoperative prevalence of delirium ranging from 16.4% to 57.6% in cohorts with prospective assessments [7,9,20,21]. A preponderance of research has indicated that preoperative delirium is associated with suboptimal short-term outcomes, including elevated inpatient mortality and prolonged hospital stays.

In our study, 4991 (23.6%) patients already had preoperative delirium upon admission. There was no relevant difference in the prevalence of delirium at admission between patients with proximal femur fractures and those with periprosthetic/peri-implant fractures (23.6%; periprosthetic/peri-implant fracture n = 294; proximal femur fracture n = 4697). During their hospital stay, 8.3% of patients with proximal femur fracture and 9.8% of patients with periprosthetic/peri-implant fracture of the patients, respectively, developed delirium. Patients with delirium received preoperative co-management by geriatricians more frequently (36.8% in patients with proximal femur fractures with pre-existing delirium vs. 26.6% without delirium; 42.4% in patients with periprosthetic/peri-implant fractures with pre-existing delirium vs. 34.2% without delirium). This approach should be extended to all patients, particularly those with an elevated risk profile. In the unadjusted analysis, in-hospital mortality was not significantly higher among patients presenting with delirium compared with those without delirium (proximal femur fracture: 8.6% vs. 4.6%; periprosthetic/peri-implant fracture: 9.6% vs. 4.1%). This statement is conclusive as delirium is strongly associated with age, comorbidity, frailty, institutionalization, and other factors that may independently influence mortality. In the proximal femur fracture group, delirium at admission was associated with higher in-hospital mortality in the unadjusted analysis. However, after adjustment for relevant confounding factors, the association between delirium at admission and mortality was no longer statistically significant. Thus, although delirium identifies a patient population at increased risk of adverse short-term outcomes, our findings do not support the conclusion that delirium itself independently increases mortality.

A similar pattern was observed in patients with periprosthetic/peri-implant fractures. Univariate analyses suggested higher mortality and higher revision rates among patients with pre-existing delirium. However, these associations were no longer statistically significant after multivariable adjustment. These findings indicate that the observed differences in outcomes may be explained, at least in part, by differences in baseline patient characteristics rather than by delirium itself. Therefore, the results should be interpreted as associations rather than evidence of an independent causal effect of delirium on mortality or revision.

The mortality rates observed in our cohort were nevertheless below those reported in a large epidemiological study from Germany. That study reported a 30-day mortality rate of 10.3% for patients treated in hospitals with orthogeriatric co-management compared with 13.4% in hospitals without such management. The difference in mortality remained evident during follow-up of up to six months [22]. However, these findings from the literature cannot be directly transferred to the present study, particularly because the present analyses indicate that the association between delirium and longer-term mortality is attenuated after adjustment for relevant patient characteristics.

Early mobilization, defined as within 24–48 h post-surgery, has been shown to be associated with improved functional outcomes and reduced hospital stay duration. A number of studies have indicated that patients who are mobilized in an early phase of recovery demonstrate a reduced occurrence of delirium [23].

The finding that patients who exhibited delirium preoperatively demonstrated reduced mobility after seven days when compared to those who did not experience delirium on admission is not unexpected. A significant proportion, amounting to 36.5% with pre-existing delirium and a proximal femur fracture and 48.4% with pre-existing delirium and a periprosthetic/peri-implant fracture, had not yet been mobilized.

The Registry for Geriatric Trauma (ATR-DGU) has also documented a 20% reduction in mortality after 120 days of early geriatric rehabilitation within an acute care facility [24].

However, due to the absence of rehabilitation potential, patients in our study returned to nursing homes with greater frequency (45.5% with delirium vs. 21.8% without delirium) than to acute geriatric care facilities, precluding the ability to draw definitive conclusions regarding this matter. This could be one reason for the shorter length of stay among patients with delirium in hip fracture patients. It is possible that these patients were transferred back to the care home earlier, rather than waiting for a place in a geriatric rehabilitation unit. However, these patients should not be denied acute geriatric care from the outset, as delirium can still be treated there while promoting mobility. There is a high probability that patients in nursing homes will not be able to be properly mobilized.

### Limitations of the Study

The findings of the present study are subject to several limitations. Firstly, it must be noted that this is a retrospective study, which inherently carries potential biases such as selection bias and confounding. The quality and completeness of the data are contingent on the accuracy of documentation, and not all variables were available for every patient, which is a common limitation in registry-based studies.

The exclusion of approximately one-third of the cases resulted in a reduction in the sample size from 32,934 to 21,127 individuals. Additionally, no adjustment for multiplicity was performed. The potential for residual confounding remains a possibility, and the heterogeneity of the delirium screening methods may have impacted the comparability of the collected data. In addition, the clinics are free to choose their own tool for delirium screening. When the Nu-desc score is not used, the questionnaire unfortunately does not record which type of delirium screening was used. There is also no information regarding the frequency of screenings during inpatient stay.

Notwithstanding, the study boasts several merits. The substantial sample size contributes to the unification of the study population, thereby reducing the impact of numerous confounding factors that can otherwise impede the analysis of treatment outcomes. Furthermore, patients were recruited from multiple centers across Germany, and all were treated within an orthogeriatric care setting, thereby enhancing the generalizability of the findings.

## 5. Conclusions

This analysis from the Registry for Geriatric Trauma (ATR-DGU) confirms that delirium is a prevalent and clinically significant complication in geriatric patients with proximal femoral fractures. At admission, nearly a quarter of all patients who underwent screening exhibited signs of delirium, underscoring the elevated vulnerability of this patient population. The comparable delirium rate between classic hip fractures and peri-implant/periprosthetic fractures indicates that the type of fracture itself is not the primary factor. Rather, patient-related factors such as age, frailty, and need for care appear to be the predominant factors.

Patients in a delirious state were found to be consistently older, frailer, and more likely to be institutionalized. This finding indicates a higher preclinical risk and limited physiological reserves.

The consequences of delirium are far-reaching. They include a higher likelihood of transfer to a nursing home. The elevated rates of intensive care observed in cases of peri-implant/periprosthetic fractures indicate the potential for more intricate post-hospitalization courses.

Despite the existence of established orthogeriatric co-management structures, delirium continues to occur with high frequency and has a significant impact on treatment success. The data suggest that existing prevention and treatment protocols require further optimization. Subgroups with heightened vulnerability, such as patients exhibiting pronounced frailty, elevated nursing care requirements, or peri-implant fractures, could benefit from enhanced preoperative assessments, structured delirium prophylaxis, and the implementation of geriatric co-care.

The results of the study indicate that delirium remains a significant risk in geriatric trauma care and serves as a crucial point of departure for enhancing clinical and functional outcomes.

## Figures and Tables

**Table 1:** Femoral neck fracture, pertrochanteric fracture, subtrochanteric fracture.

	Delirium	No Delirium	*p*-Value
**Age (years)**			<0.001
- Median (IQR)	86 (82–91)	84 (80–88)	
**Gender (Female)**	69.2% (n = 3245)	70.6% (n = 10,714)	0.06
**ASA**			<0.001
- Median (IQR)	3.0 (3.0–3.0)	3.0 (2.0–3.0)	
**ISAR**			<0.001
- ≥2	93.2% (n = 3254)	75.4% (n = 8563)	
**CFS**			<0.001
- Median (IQR)	6.0 (5.0–7.0)	5.0 (3.0–6.0)	
**Place of residence**			<0.001
- At home	60.6% (n = 2815)	86.0% (n = 12,898)	
- Nursing home	38.4% (n = 1783)	13.4% (n = 2009)	
- Other	0.9% (n = 45)	0.6% (n = 89)	
**Mobility**			<0.001
- Independently	22.7% (n = 969)	40.6% (n = 5852)	
- With the help of tools	46.1% (n = 1967)	46.0% (n = 6622)	
- No or hardly any ability to walk	31.1% (n = 1327)	13.4% (n = 1934)	
**Preoperative co-treatment by geriatricians**	36.8% (n = 1707)	26.6% (n = 3970)	<0.001
**Type of fracture**			0.082
- Femoral neck	47.6% (n = 2236)	49.03% (n = 7446)	
- Pertrochanteric	48.6% (n = 2283)	46.8% (n = 7111)	
- Subtrochanteric	3.8% (n = 178)	4.1% (n = 629)	
**Time to surgery (h)**			0.008
- Median (IQR)	16.3 (7.7–22.5)	15.9 (7.0–22.3)	
**Postoperative treatment at ICU**	28.8% (n = 1331)	27.3% (n = 4095)	0.042
**Mobility 7 days after surgery**			<0.001
- Independently	0.8% (n = 33)	0.6% (n = 81)	
- With the help of tools	62.8% (n = 2765)	83.2% (n = 12,032)	
- No or hardly any ability to walk	36.5% (n = 1608)	16.2% (n = 2343)	
**Non-surgical complications**	35.9% (n = 1634)	37.5% (n = 5572)	0.056
**Need for surgical revision**	2.51% (n = 112)	2.94% (n = 431)	0.149
**Mortality**	8.6% (n = 403)	4.6% (n = 703)	<0.001
**Place of discharge**			<0.001
- Home	18.9% (n = 806)	31.7% (n = 4572)	
- Rehabilitation	0.8% (n = 34)	4.2% (n = 602)	
- Geriatric rehabilitation	31.9% (n = 1364)	39.4% (n = 5684)	
- Other hospital	3.1% (n = 134)	3.0% (n = 436)	
- Nursing home	45.3% (n = 1935)	21.8% (n = 3143)	
**Length of stay (d)**			<0.001
- Median (IQR)	13.1 (8.1–20.0)	16.1 (9.1–21.1)	<0.001
**Mortality (120 d follow-up)**	17.3% (n = 192)	8.9% (n = 342)	<0.001

Abbreviations: IQR = interquartile range; ASA = American Society of Anesthesiologists; ISAR = Identification of seniors at risk; CFS = Clinical Frailty Scale; h = hour; ICU = Intensive Care Unit; d = day. Data are presented as median (IQR) or n (%). Percentages are based on available cases for each variable. Missing values were excluded from the respective analyses.

**Table 2:** Patients with delirium, femoral neck fracture, pertrochanteric fracture, subtrochanteric fracture—multivariate analysis.

Logistic Regressions:				
***Postoperative treatment at ICU***				
**Number of** **observations**	**OR**	**95% lower CI**	**95% upper CI**	***p* ** **value**
13,141	0.84	0.75	0.94	0.002
***Non-surgical complications***				
**Number of** **observations**	**OR**	**95% lower CI**	**95% upper CI**	***p* ** **value**
13,010	1.24	1.12	1.38	<0.001
**Need for surgical revision**				
**Number of** **observations**	**OR**	**95% lower CI**	**95% upper CI**	***p* ** **value**
12,934	0.89	0.65	1.2	0.447
***Mortality***				
***Number of*** ***observations***	***OR***	***95% lower CI***	***95% upper CI***	***p value***
13,231	1.18	0.97	1.44	0.091
***Mortality (120 d follow-up)***				
**Number of** **observations**	**OR**	**95% lower CI**	**95% upper CI**	***p* ** **value**
3497	1.03	0.79	1.36	0.818
**Linear regression**				
***Time to surgery (h)***				
**Number of** **observations**	**percentage change (95% CI)**	**95% lower CI**	**95% upper CI**	***p* ** **value**
12,965	−0.32	−4.37	3.89	0.878
***Length of stay (d)***				
**Number of** **observations**	**percentage change (95% CI)**	**95% lower CI**	**95% upper CI**	***p* ** **value**
13,182	−12.29	−14.96	−19.54	<0.001

Abbreviations: OR = odds ratio; CI = confidence interval; ICU = Intensive Care Unit. Number of observations refers to complete cases included in the respective regression model. Differences in sample size are due to missing values in outcome variables and/or covariates.

**Table 3:** Periprosthetic and peri-implant fracture.

	Delirium	No Delirium	*p*-Value
**Age (years)**			<0.001
- Median (IQR)	86 (83–90)	84 (79–88)	
**Gender (Female)**	71.4% (n = 210)	72.0% (n = 683)	0.915
**ASA**			<0.001
- Median (IQR)	3.0 (3.0–3.0)	3.0 (3.0–3.0)	
**ISAR**			<0.001
- ≥2	91.6% (n = 197)	78.9% (n = 565)	
**CFS**			<0.001
**-** Median (IQR, median)	6.0 (5.0–7.0)	4.0 (3.0–6.0)	
**Place of residence**			<0.001
- At home	69.6% (n = 201)	88.6% (n = 836)	
- Nursing home	28.7% (n = 83)	10.4% (n = 98)	
- Other	1.7% (n = 5)	1.1% (n = 10)	
**Mobility**			<0.001
**- Independently**	19.9% (n = 52)	32.6% (n = 292)	
**- With the help of tools**	48.1% (n = 126)	54.7% (n = 490)	
**- No or hardly any ability to walk**	32.1% (n = 84)	12.7% (n = 114)	
**Preoperative** **co- treatment by geriatricians**	42.4% (n = 122)	34.2% (n = 318)	0.014
**Type of fracture**			0.028
- Periprosthetic	83.7% (n = 246)	88.7% (n = 843)	
- Peri- implant	16.3% (n = 48)	11.3% (n = 107)	
**Time to surgery (h)**			0.335
- Median (IQR)	38.4 (19.3–76.4)	40.7 (20.4–85.0)	
**Postoperative treatment at ICU**	50.7% (n = 147)	40.4% (n = 379)	0.002
**Mobility 7 days after surgery**			<0.001
- Independently	0.36% (n = 1)	0.22% (n = 2)	
- With the help of tools	51.3% (n = 143)	69.7% (n = 632)	
- No or hardly any ability to walk	48.4% (n = 135)	30.1% (n = 273)	
**Non-surgical complications**	39.3% (n = 112)	39.8% (n = 368)	0.938
**Need for surgical revision**	9.6% (n = 25)	5.7% (n = 52)	0.039
**Mortality**	9.6% (n = 28)	4.1% (n = 39)	<0.001
**Place of discharge**			<0.001
- Home	17.1% (n = 45)	24.2% (n = 220)	
- Rehabilitation	2.3% (n = 6)	4.7% (n = 43)	
- Geriatric rehabilitation	28.9% (n = 76)	36.7% (n = 334)	
- Other hospital	5.7% (n = 15)	5.0% (n = 45)	
- Nursing home	46.0% (n = 121)	29.4% (n = 267)	
**Length of stay (d)**			0.138
- Median (IQR)	16.1 (11.1–24.1)	17.1 (12.1–24.1)	
**Mortality (120 d follow-up)**	17.3% (n = 201)	8.9% (n = 371)	<0.001

Abbreviations: IQR = interquartile range; ASA = American Society of Anesthesiologists; ISAR = Identification of seniors at risk; CFS = Clinical Frailty Scale; h = hour; ICU = Intensive Care Unit; d = day. Data are presented as median (IQR) or n (%). Percentages are based on available cases for each variable. Missing values were excluded from the respective analyses.

**Table 4:** Patients with delirium and periprosthetic and peri-implant fracture—multivariate analysis.

Logistic Regressions:				
***Postoperative treatment at ICU***				
**Number of** **observations**	**OR**	**95% lower CI**	**95% upper CI**	***p* ** **value**
840	1.21	0.80	1.81	0.367
***Non-surgical complications***				
**Number of** **observations**	**OR**	**95% lower CI**	**95% upper CI**	***p* ** **value**
829	1.49	0.99	2.25	0.055
***Need for surgical revision***				
**Number of** **observations**	**OR**	**95% lower CI**	**95% upper CI**	***p* ** **value**
804	1.69	0.82	3.48	0.155
***Mortality***				
**Number of** **observations**	**OR**	**95% lower CI**	**95% upper CI**	***p* ** **value**
844	1.38	0.64	2.96	0.407
***Mortality (120 d follow-up)***				
**Number of** **observations**	**OR**	**95% lower CI**	**95% upper CI**	***p* ** **value**
3781	0.17	0.03	1.12	0.066
***Linear regression***				
***Time to surgery (h)***				
**Number of** **observations**	**percentage change (95% CI)**	**95% lower CI**	**95% upper CI**	***p* ** **value**
836	−27.95	−41.75	−10.88	0.003
***Length of stay (d)***				
**Number of** **observations**	**percentage change (95% CI)**	**95% lower CI**	**95% upper CI**	***p* ** **value**
836	−9.11	−18.34	1.18	0.081

Abbreviations: OR = odds ratio; CI = confidence interval; ICU = Intensive Care Unit. Number of observations refers to complete cases included in the respective regression model. Differences in sample size are due to missing values in outcome variables and/or covariates.

**Table 5:** Delirium during hospitalization.

	Delirium	No Delirium	*p*-Value
**Mortality**	7.0% (n = 93)	4.4% (n = 661)	<0.001
**Place of discharge**			<0.001
- Home	21.4% (n = 265)	32.2% (n = 4645)	
- Rehabilitation	1.9% (n = 23)	4.4% (n = 635)	
- Geriatric rehabilitation	36.5% (n = 451)	39.31 (n = 5669)	
- Other hospital	3.4% (n = 42)	3.1% (n = 448)	
- Nursing home	36.8% (n = 455)	21.0% (n = 3024)	
**Mobility follow-up**			<0.001
- Independently	7.1% (n = 21)	16.6% (n = 569)	
- Walking stick	9.1% (n = 27)	15.4% (n = 528)	
- 2 crutches or walker	47.6% (n = 141)	47.0% (n = 1615)	
- Some ability to walk in the flat	17.6% (n = 52)	13.9% (n = 477)	
- No walking ability	18.6% (n = 55)	7.2% (n = 247)	
**Mortality follow-up**	13.9% (n = 48)	8.5% (n = 323)	<0.001

Data are presented as n (%). Percentages are based on available cases for each variable. Missing values were excluded from the respective analyses.

**Table 6:** Delirium during hospitalization—multivariate analysis.

Logistic Regressions:				
***Mortality***				
**Number of** **observations**	**OR**	**95% lower CI**	**95% upper CI**	***p* ** **value**
11,018	1	0.74	1.36	0.983
***Mortality (120 d follow-up)***				
**Number of** **observations**	**OR**	**95% lower CI**	**95% upper CI**	***p* ** **value**
2957	1	0.64	1.56	0.99
***Need for surgical revision (120 d follow-up)***				
**Number of** **observations**	**OR**	**95% lower CI**	**95% upper CI**	***p* ** **value**
2625	1.72	0.91	3.26	0.097

Abbreviations: OR = odds ratio; CI = confidence interval. Number of observations refers to complete cases included in the respective regression model. Differences in sample size are due to missing values in outcome variables and/or covariates.

## Data Availability

The original contributions presented in this study are included in the article. Further inquiries can be directed to the corresponding author.
